# Cauda equina nerve root enhancement in adult intestinal toxemia botulism

**DOI:** 10.1186/s12883-025-04365-4

**Published:** 2025-08-22

**Authors:** Ruiji Jiang, Benjamin Beland, Vinil Shah, Min Kang, Douglas B. Pet

**Affiliations:** 1 Department of Neurology, University of California, San Francisco, USA; 2 Division of Neurology, Department of Medicine, University of Ottawa, Ottawa, Canada; 3 Department of Neuroradiology, University of California, San Francisco, USA

**Keywords:** Botulism, Adult intestinal toxemia botulism, AITB, Nerve root enhancement, Abnormal imaging, Magnetic resonance imaging, MRI

## Abstract

**Background:**

Adult botulism is a rare, life-threatening condition typically caused by exposure to preformed botulinum neurotoxin (BoNT). Acute intestinal toxemia botulism (AITB) is an uncommon subtype resulting from colonization of *Clostridium botulinum* in the intestines. Diagnosis is made by detecting BoNT in the patient’s blood, stool, or gastric fluid. AITB is confirmed when *C. botulinum* is isolated in culture. Electrodiagnostic studies may support the diagnosis, while imaging—when performed—is generally used to exclude alternative conditions.

**Case presentation:**

A 74-year-old man presented with acute dysarthria and ophthalmoparesis, which rapidly progressed to quadriparesis and respiratory failure requiring intubation. Magnetic resonance imaging (MRI) revealed thickening and enhancement of the cauda equina nerve roots. Due to high clinical suspicion for botulism, heptavalent botulinum antitoxin was administered. Intravenous immunoglobulin was also given, as the imaging findings raised concern for an alternative diagnosis of Guillain-Barré syndrome (GBS). Blood and stool samples later tested positive for BoNT type A, and *C. botulinum* was isolated from the stool, confirming AITB. The patient experienced a gradual but prolonged recovery of motor function following treatment.

**Conclusions:**

Botulism in both infants and adults is not typically associated with abnormal neuroimaging findings. To our knowledge, this is the first reported case of cauda equina nerve root thickening and enhancement on MRI in AITB—or in adult botulism more broadly. We outline the differential diagnosis, pathophysiology, and treatment of botulism. This case underscores that abnormal neuroimaging should not delay prompt empiric treatment for botulism when clinical suspicion is high.

## Background

Adult botulism is a rare life-threatening condition caused by exposure to preformed botulinum neurotoxin (BoNT) or, rarely, in situ toxin production due to *Clostridium botulinum* bacterial colonization of a wound (wound botulism) or the intestine [adult intestinal toxemia botulism (AITB)]. Symptoms result from presynaptic failure at the neuromuscular junction (NMJ) and typically progress rapidly from nausea, to acute bulbar and pupillary dysfunction, to descending paralysis and respiratory failure [1–5]. We report a patient with AITB associated with cauda equina nerve root thickening and gadolinium enhancement on magnetic resonance imaging (MRI). To our knowledge, these are the first reported radiographic findings of this type associated with adult botulism. We describe the natural history, diagnosis, and treatment of adult botulism and AITB.

### Case presentation

A 74-year-old man with osteoarthritis and alcohol use disorder presented with double vision and slurred speech after a nap (Day 0). By Day 1, double vision had resolved but the slurring persisted, prompting ED evaluation where he was tachypneic but neurologically intact. Labs, including blood counts, chemistries, troponin, lactate, ethanol level, and urine toxicology, were unremarkable. Computed tomography (CT) of the brain with angiography was normal. Within hours, dysarthria worsened, oropharyngeal secretions accumulated, and aspiration occurred, leading to hypoxia and intubation.

Examination several hours later in the intensive care unit (ICU) showed intact mental status, dilated and sluggishly reactive pupils, bifacial weakness, near-complete ophthalmoplegia (slight preservation of left eye adduction), intact corneal, gag, and cough reflexes, full shoulder strength, decreased tone, severe symmetric proximal weakness, head drop, mild length-dependent sensory deficits, and absent reflexes. Babinski reflexes were equivocal. MRI brain showed mild subcortical white matter T2/fluid attenuated inversion recovery (FLAIR) hyperintensities consistent with chronic microvascular disease. Cerebrospinal fluid (CSF) revealed normal white blood cell (WBC) count and glucose with mildly elevated protein (52 mg/dL).

On Day 3, he was transferred to our tertiary center where he remained alert, communicating via thumbs-up/down. Pupils were 4 mm with minimal right-sided reactivity. The neurologic exam was unchanged. Of note, no fatiguability was noted on repetitive testing of biceps flexion. and reflexes remained absent throughout despite Jendrassik maneuvers and skin exam revealed no rashes or ticks. The constellation of acute bulbar dysfunction, flaccid weakness, and respiratory failure suggested a fulminant neuromuscular process localizing to the neuromuscular junction, nerve roots, nerves, and/or muscles. The patient’s descending paralysis, mydriasis, and rapid deterioration favored botulism, however Guillain-Barré syndrome (GBS) Miller-Fisher variant (given areflexia, sensory findings of unknown chronicity, and elevated CSF protein) and other autoimmune, paraneoplastic, infectious, and toxicologic etiologies were also considered. Comprehensive workup was initiated (Table 1). He was empirically treated on Day 3 with both equine Botulinum anti-toxin (BAT) Type A and initiated on intravenous immunoglobulin (IVIg) 2 g/kg over 5 days for possible GBS.

**Table 1:** Key diagnostic work-up

Specimen source:	Test (results within normal range, unless otherwise specified)
Serum:	Bacterial cultures, HIV Ag/Ab, Amphiphysin, ANNA-1/3, AGNA-1, AP3B2, CRMP5 CASPR2, Contactin-1, GFAP, GM1 IgG/IgM, GD1b IgG/IgM, Gd1b IgG/GM, MAG IgM, WNV IgG, AChR IgG, igLON5, LG1, MuSK Ab, MAG, Neurofascin, LRP4, HIV Ag/Ab, botulinum toxin type-A positive, botulinum toxin mouse bioassay positive,
Urine:	Toxicology screen, culture
CSF:	Cell count, protein (54 mg/dl), glucose (102 mg/dl), gram stain/bacterial culture, IgG index, oligoclonal bands, WNV IgG/IgM, VZV IgG/IgM, VDRL, B. Burgdorferi Ab, Meningitis PCR panel (H. Influenza, Group B Strep, Strep pneumo, N. Meningitidis, E. Coli K1, CMV, Listeria, Enterovirus, HSV1/HSV2, HHSV6 PCR, Parechovirus, VZV, Cryptococcus), Paraneoplastic IgG panel (ANNA-1, ANNA-2, PCCA-1, PCCA-Tr)
Stool:	Culture positive C. botulinum, C. Botulinum toxin negative

Normal values CSF Protein (15–45 mg/dL), CSF Glucose (40–70 mg/dL), CSF Nucleated Cells (0–6/uL), CSF RBC (0/uL)

**Fig. 1:**
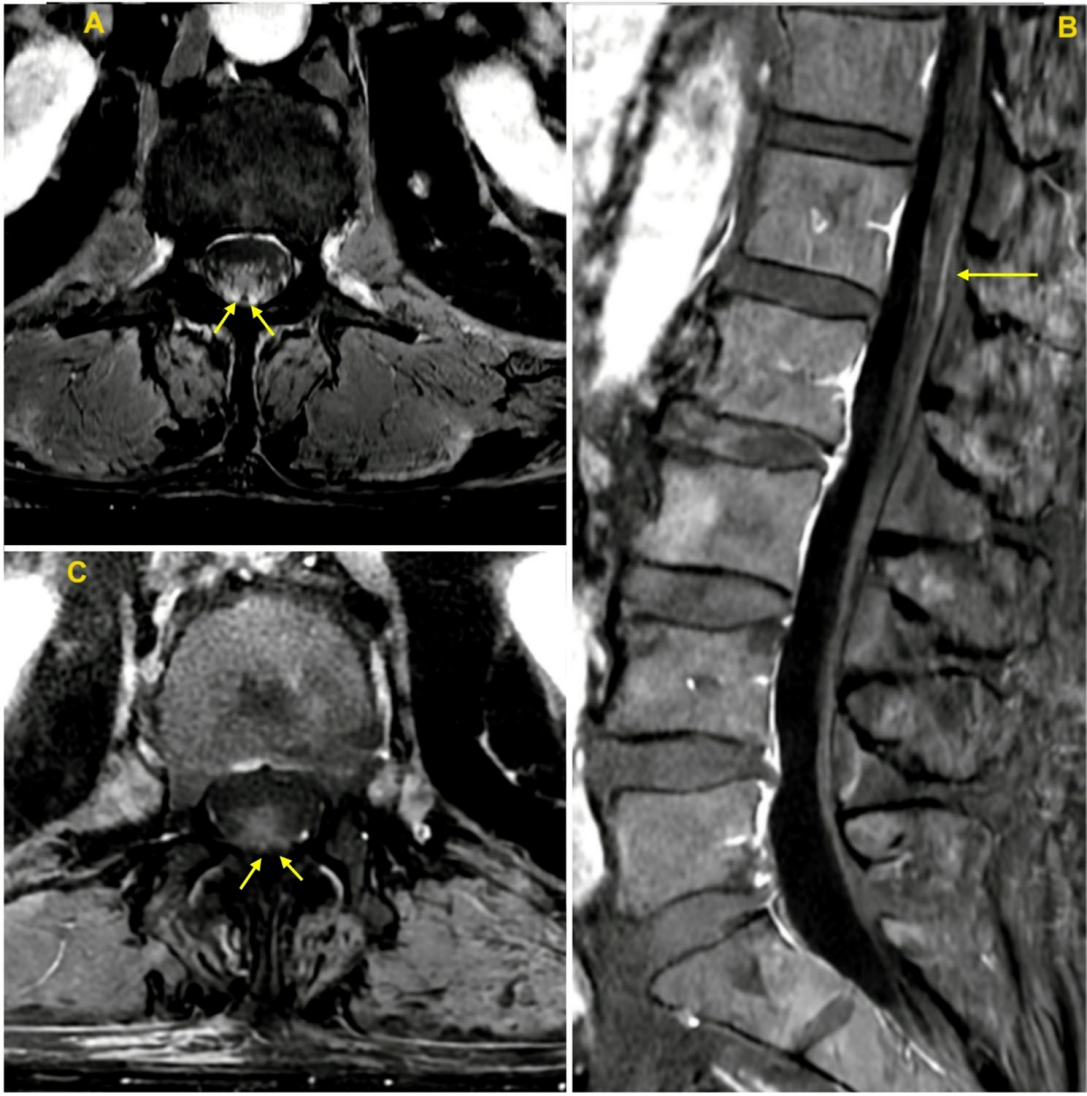
Initial and delayed spinal imaging (**A**) Axial and (**B**)Sagittal T1 post-gadolinium MRI obtained on day 5 of illness showing thickening and avid enhancement of the dorsal cauda equina roots (yellow arrows) at the level of the conus medullaris. (**C**) Axial T1 post- gadolinium MRI obtained 3 months after illness showing persistent, though markedly decreased, cauda equina root thickening and enhancement (yellow arrows)

The following day, previously absent patellar deep tendon reflexes (DTR) were elicited easily (2 + left, 1 + right). Early return and persistence of DTRs was recognized to be unusual for GBS, and, along with increasingly dilated and poorly reactive pupils, further elevated concerns for botulism. Unexplained DTR abnormalities further prompted spinal MRI to assess for an upper motor neuron processes, and this showed normal spinal cord and mild enhancement and thickening of the dorsal cauda equina nerves roots near the level of the conus (Fig. 1). This finding was not an expected feature of the leading clinical diagnosis of botulism and carried a broad radiologic differential including GBS, chronic inflammatory demyelinating polyneuropathy (CIDP), infection (e.g., Lyme disease, tuberculosis), autoimmune disorders (e.g., neurosarcoidosis), or malignancy (e.g., neurolymphomatosis).

Nerve conduction studies (NCS) on Day 3 revealed reduced amplitudes of CMAPs (median, peroneal), preserved sensory nerve action potentials (ulnar, radial, sural), slowed tibial/peroneal motor and sural sensory conduction, and absent conduction block or temporal dispersion. The absence of decrement/increment on repetitive ulnar nerve stimulation (3 Hz/50Hz) made myasthenia gravis less likely. Minimal F-wave latencies were prolonged for the left median and tibial nerves and absent in the left ulnar and peroneal nerves. Needle EMG of the deltoid showed short-duration, low amplitude motor unit action potentials (MUAP) with early recruitment, and the biceps, triceps and tibialis anterior showed morphologically normal MUAPs with mild-to-moderately reduced recruitment. There were no acute denervation potentials. In addition to an incidental median neuropathy at the left wrist, the presence of reduced CMAPs, persevered SNAPs, and myopathic features supported a pre-synaptic NMJ disorder consistent with botulism. However, additional features raised the question of a superimposed neuropathy with axonal and demyelinating features. Due to the marked motor predominance, chronic polyneuropathy (e.g., alcohol-related) was felt to be less likely. F-wave abnormalities were difficult to interpret in the setting of potential ICU artifact, however, paired with other findings and the patient’s imaging, a proximal inflammatory process like early GBS, could not be excluded.

On Day 5, serum testing by California Department of Public Health (DPH) returned positive for BoNT type A, confirming a diagnosis of botulism. Mouse bioassay testing of the patient’s serum samples resulted in the death of 4 out of 4 mice in less than 12 h, which suggested high levels of circulating BoNT. A careful skin exam showed no wounds that could be a source of wound botulism. Stool culture subsequently grew BoNT type A-producing *C. botulinum*, confirming AITB. Thorough search by DPH of the patient’s residence, as well as sample collection and thorough analysis isolated BoNT type A-producing *C. botulinum* from a tumbler containing a homemade alcoholic beverage, which the patient had consumed on the day of symptom onset, as well as the surrounding soil, making it the most likely inoculation source.

Over the ensuing months, the patient’s motor function gradually improved. He required tracheostomy and gastrostomy for persistent respiratory insufficiency and dysphagia, and experienced deconditioning and orthostatic hypotension treated with midodrine. At 2 months, he could propel a wheelchair but struggled with head control and secretions. Subsequent hospitalization for aspiration pneumonia and septic shock further delayed recovery. At 3 months, MRI showed improving but persistent cauda equina enhancement (Fig. 1). By 5 months, he could transition from lying to sitting with assistance, could stand using a lift device, and began walk training. He was later re-hospitalized for catheter-associated sepsis.

## Discussion

Botulism is rare with only about 110 cases reported annually in the United States. Infant botulism occurs due to ingestion and intestinal colonization of *C. botulinum* spores, which in turn, produce BoNT. Most adult disease is acquired through ingestion of preformed neurotoxin, or, less commonly, toxin production in an infected wound. Iatrogenic disease has also rarely occurred due to BoNT use for cosmetic or medical indications. Botulism due to intestinal colonization in adults, denoted AITB, occurs in less than 1% of adult cases and has been reported in association with 6 out of 7 botulism toxin subtypes (A-F). AITB is typically associated with pre-existing immunosuppression, abnormal gastrointestinal anatomy (e.g., prior colon resection, short bowel syndrome, post-jejunoileal bypass, Meckel’s diverticulum), mucosal dysregulation (e.g., inflammatory bowel disease, achlorhydria), and/or dysbiosis (e.g., recent systemic antibiotic therapy) which can potentiate colonization by disrupting local gut flora [6–9]. AITB has been reported in the absence of risk factors, as in the current case [5, 7, 8, 10–13].

Due to botulism’s acute and often fulminant onset, diagnosis and appropriate treatment hinge on early recognition of signs and symptoms, such as bulbar dysfunction (often including dilated and poorly reactive pupils), descending flaccid paralysis, and neuromuscular respiratory failure. The most sensitive test and laboratory gold standard for diagnosing botulism is mouse lethality bioassay, in which mice are injected with a patient’s serum sample and observed for signs of disease, including fuzzy hair, weakness, and respiratory failure. Additional enzyme-linked immunoassays are used to differentiate toxin subtypes though sensitivity is significantly lower [14]. Confirmatory results of botulism testing are inevitably delayed beyond the acute period during which prompt antitoxin administration is critical.

While often non-specific, electrodiagnostic findings may support the diagnosis of botulism and can help to exclude mimics [15]. Typical features associated with presynaptic NMJ disorders, like botulism, include reduced CMAP amplitudes with preserved sensory responses. Distal motor latencies and motor conduction velocities may be normal or slightly slowed The apparent slowing of conduction velocity, a finding typically associated with demyelination, may instead be a physiological artifact in this case. When NMJ transmission fails at the synapses of the fastest-conducting motor fibers, the resulting CMAP is initiated by slower fibers, resulting in prolonged latency or slow conduction velocities. A similar rationale is given for the slowing of nerve conductions observed in axonal neuropathies [16]. The most characteristic RNS finding is an incremental response (facilitation) after brief, maximal exercise or with high-frequency RNS (30-50hz). Modest decrement of CMAP amplitudes may be observed with low-frequency (3 Hz) repetitive nerve stimulation (RNS), but this is less pronounced than in myasthenia gravis in general. Late responses and sensory NCS are typically normal. Impaired neuromuscular transmission can lead to incomplete activation of muscle fibers, producing motor unit potentials with a myopathic appearance and an early recruitment pattern. In cases of more severe transmission block, there may be functional loss of motor units, resulting in reduced recruitment. The mixed recruitment patterns observed in this case likely reflect varying degrees of partial to complete motor unit blockade, compounded by a superimposed neurogenic process, such as the diffuse radiculopathies seen on MRI. It is important to note that electrodiagnostic findings can vary, making interpretation challenging, particularly in early disease [15, 16]. 

Brain and spinal imaging, when reported, is typically normal in botulism and primarily serves to exclude alternative diagnoses [1, 4, 17]. To our knowledge, the only other report of abnormal MRI findings was a case of infantile botulism associated with symmetric cervical nerve root enhancement, without reported nodularity or thickening, and subtle diffusion restriction in the splenium, dorsal pons, and bilateral optic radiations. This patient responded rapidly to treatment with botulism immunoglobulin and, similarly to our case, BoNT type A was identified [18]. The true prevalence of nerve root and/or other abnormalities on MRI may be higher than reported in the botulism literature, given that neuro-imaging, much less, delayed imaging, is not routinely obtained [1, 4, 17]. 

As BoNT is understood to act primarily at the presynaptic nerve terminal, a mechanism for nerve root involvement is not clear [19]. There is mounting evidence that botulism pathophysiology extends beyond the NMJ. For example, a study in mice demonstrated remnants of SNAP25, a key protein cleaved by BoNT at the presynaptic terminal, in sciatic nerve axons, dorsal root ganglia, and spinal cord [20]. Others have proposed retrograde axonal transport of BoNT, although mechanisms have not been well established [21, 22]. While little can be speculated based on two cases, the presence of true clostridial infection in both our adult patient and, presumably, the previously reported infant raises the possibility that the observed root enhancement may have represented an inflammatory response, perhaps analogous to that seen in post-infectious GBS. Clostridial infectious radiculitis has not been reported, and bland cerebrospinal fluid (CSF) profiles in both AITB cases suggest this to be unlikely.

The possibility of co-occurring GBS was considered as an explanation for our patient’s imaging findings and mixed demyelinating features on NCS [23, 24]. Given that this diagnosis of GBS could not be excluded, and due to early diagnostic uncertainty and rapid clinical worsening, a full course of empiric IVIg was completed out of an abundance of caution. Given the patient’s clinical course (including persistently present DTRs), positive confirmatory testing for botulism, and findings on EMG/NCS that could be consistent with early GBS, we ultimately felt that an additional diagnosis of GBS was unlikely.

Treatment for non-infantile, as well infantile cases felt to be foodborne, is equine heptavalent BAT, which acts against 7 of 8 known toxin subtypes ideally administered in the first 48 h for maximal effect. Infantile botulism due to bacterial colonization is treated with human-derived intravenous botulism immune globulin (known as BIG-IV or BabyBIG) [25, 26]. Antibiotics are currently only recommended for wound botulism and there is no current recommendation to treat AITB with BIG or antibiotics. Antibiotics are avoided, however, in cases of infantile botulism due to a theoretical risk that bacteriolysis may augment toxin release and worsen symptoms [27]. While no formal studies have investigated their efficacy in AITB, vancomycin and metronidazole have been reported as adjunctive treatments aimed at clearing intestinal colonization [7, 28]. Supportive care in the ICU is crucial to preventing morbidity and mortality. Approximately 46–70% of patients with botulism require intubation and mechanical ventilation, and patients can also experience marked dysautonomia, including heart rate and blood pressure lability as well as anhidrosis [29, 30]. Even with timely BAT treatment, recovery typically spans 30–100 days, though potentially longer [7, 25, 29]. Due to AITB’s rarity, it is unclear if recovery rates differ, though some reports, including ours, describe complicated and prolonged courses [7]. One AITB case report described recurrence of descending paralysis on days 23–25 associated with recurrent detection of botulinum toxin in the stool, despite initial stabilization and improvement following BAT administration on day 13 [28]. 

## Conclusion

To our knowledge, this is the first reported case of adult botulism associated with cauda equina nerve root enhancement on MRI. The etiology of radiographic nerve root involvement in botulism remains unclear. However, this case supports the notion that MRI abnormalities such as these should not dissuade from or delay prompt empirical treatment for botulism in the appropriate clinical context.

## Data Availability

No datasets were generated or analysed during the current study.

## Abbreviations

Ab: Antibody
AFB: Acid-fast bacillus
Ag: Antigen
AGNA-1: Anti-glial/neuronal nuclear antibody-type 1
AITB: Adult intestinal toxemia botulism
ANNA: Anti-nuclear neuronal antibody
AP3B2: AP-3 Complex subunit beta-2
BAT: Botulinum anti-toxin
BoNT: Botulinum neurotoxin
CASPR2: Contactin-associated protein-like 2
CPK: Creatinine kinase
CRMP5: Collapsin response mediator protein 5
CMAP: Compound motor actional potential
CSF: Cerebrospinal fluid
CT: Computerized tomography
ED: Emergency department
EMG: Electromyogram
GFAP: Glial fibrillary acidic protein
Ig: Immunoglobulin
IGRA: Interferon alpha release assay
igLON5: Immunoglobulin-like cell adhesion molecule
GQ1B: Ganglioside Q1b
GD1B: Ganglioside D1B
GM1: Monosialotetrahexosylganglioside
GM1B: Monosialotetrahexosylganglioside-B
HCV: Hepatitis C virus
HBV: Hepatitis B virus
HHSV6: Human herpes virus 6
HIV: Human immunodeficiency virus
HSV: Herpes Simplex Virus
LG1: Leucine-rich, glioma inactivated 1 gene
LRP4: Low-density lipoproteinreceptor-related protein4
MMA: Methylmalonic acid
mNGS: Metagenomic next-generation sequencing
MAG: Myelin associated glycoprotein
MUAP: Motor unit action potential
MG: Myasthenia gravis
MUSK: Muscle specific kinase
MRI: Magnetic resonance imaging
NIF IFA: Neuronal intermediate filament immunofixation assay
NMJ: Neuromuscular junction
PCA/PCCA: Propionyl-CoA carboxylase subunit alpha
PCR: Polymerase chain reaction
RPR: Rapid plasma regain
SSA/B: Sjögren's-syndrome-related antigen A and B
VDRL: Venereal disease research laboratory
VZV: Varicella zoster virus
WNV: West nile virus

## References

[1] Sobel J. Botulism. Clin Infect Dis. 2005;41(8):1167–73. 16163636 10.1086/444507

[2] Jeffery IA, Karim S. Botulism. In: StatPearls [Internet]. Treasure Island: StatPearls Publishing; 2024. [Cited 2024 Nov 9]. Available from: http://www.ncbi.nlm.nih.gov/books/NBK459273/. Accessed 9 Nov 2024.

[3] Woodruff BA, Griffin PM, McCroskey LM, Smart JF, Wainwright RB, Bryant RG, et al. Clinical and laboratory comparison of botulism from toxin types A, B, and E in the united states, 1975–1988. J Infect Dis. 1992;166(6):1281–6. 1431246 10.1093/infdis/166.6.1281

[4] Chatham-Stephens K, Fleck-Derderian S, Johnson SD, Sobel J, Rao AK, Meaney-Delman D. Clinical features of foodborne and wound botulism: a systematic review of the literature, 1932–2015. Clin Infect Dis. 2017;66(suppl1):S11-6. 29293923 10.1093/cid/cix811

[5] Chia JK, Clark JB, Ryan CA, Pollack M. Botulism in an adult associated with Food-Borne intestinal infection with Clostridium botulinum. N Engl J Med. 1986;315(4):239–41. 3523248 10.1056/NEJM198607243150407

[6] Bartlett JC. Infant botulism in adults. N Engl J Med. 1986;315(4):254–5. 3523249 10.1056/NEJM198607243150411

[7] Harris RA, Anniballi F, Austin JW. Adult intestinal toxemia botulism. Toxins (Basel). 2020;12(2): 81. 31991691 10.3390/toxins12020081 PMC7076759

[8] McCroskey LM, Hatheway CL. Laboratory findings in four cases of adult botulism suggest colonization of the intestinal tract. J Clin Microbiol. 1988;26(5):1052–4. 3290234 10.1128/jcm.26.5.1052-1054.1988 PMC266519

[9] Griffin PM, Hatheway CL, Rosenbaum RB, Sokolow R. Endogenous antibody production to botulinum toxin in an adult with intestinal colonization botulism and underlying Crohn’s disease. J Infect Dis. 1997;175(3):633–7. 9041335 10.1093/infdis/175.3.633

[10] McCroskey LM, Hatheway CL, Woodruff BA, Greenberg JA, Jurgenson P. Type F botulism due to neurotoxigenic clostridium baratii from an unknown source in an adult. J Clin Microbiol. 1991;29(11):2618–20. 1774272 10.1128/jcm.29.11.2618-2620.1991 PMC270386

[11] Fenicia L, Franciosa G, Pourshaban M, Aureli P. Intestinal toxemia botulism in two young people, caused by clostridium Butyricum type E. Clin Infect Dis. 1999;29(6):1381–7. 10585782 10.1086/313497

[12] Fenicia L, Anniballi F, Aureli P. Intestinal toxemia botulism in Italy, 1984–2005. Eur J Clin Microbiol Infect Dis. 2007;26(6):385–94. 17516104 10.1007/s10096-007-0301-9

[13] Freund B, Hayes L, Rivera-Lara L, Sumner C, Chaudhry V, Chatham‐Stephens K, et al. Adult intestinal colonization botulism mimicking brain death. Muscle Nerve. 2017;56(4). [Cited 2024 Nov 9]. Available from:10.1002/mus.25689. 10.1002/mus.25689 28500638

[14] Centurioni DA, Egan CT, Perry MJ. Current developments in diagnostic assays for laboratory confirmation and investigation of botulism. J Clin Microbiol. 2022;60(4):e0013920. 34586891 10.1128/jcm.00139-20 PMC9020338

[15] Rao AK, Sobel J, Chatham-Stephens K, Luquez C. Clinical guidelines for diagnosis and treatment of botulism, 2021. MMWR Recomm Rep. 2021;70(2):1–30. 33956777 10.15585/mmwr.rr7002a1 PMC8112830

[16] Chung T, Prasad K, Lloyd TE. Peripheral neuropathy: clinical and electrophysiological considerations. Neuroimaging Clin N Am. 2014;24(1):49–65. 24210312 10.1016/j.nic.2013.03.023 PMC4329247

[17] Gupta A, Sumner CJ, Castor M, Maslanka S, Sobel J. Adult botulism type F in the United States, 1981–2002. Neurology. 2005;65(11):1694–700. 16344510 10.1212/01.wnl.0000187127.92446.4c

[18] Good RJ, Messacar K, Stence NV, Press CA, Carpenter TC. Abnormal neuroimaging in a case of infant botulism. Front Pediatr. 2015;3:108. 26697417 10.3389/fped.2015.00108 PMC4676149

[19] Simpson LL. Identification of the major steps in botulinum toxin action. Annu Rev Pharmacol Toxicol. 2004;44:167–93. 14744243 10.1146/annurev.pharmtox.44.101802.121554

[20] Rawson AM, Dempster AW, Humphreys CM, Minton NP. Pathogenicity and virulence of clostridium botulinum. Virulence. 2023;14(1):2205251. 37157163 10.1080/21505594.2023.2205251 PMC10171130

[21] Restani L, Giribaldi F, Manich M, Bercsenyi K, Menendez G, Rossetto O, et al. Botulinum neurotoxins A and E undergo retrograde axonal transport in primary motor neurons. PLoS Pathog. 2012;8(12):e1003087. 23300443 10.1371/journal.ppat.1003087 PMC3531519

[22] Antonucci F, Rossi C, Gianfranceschi L, Rossetto O, Caleo M. Long-distance retrograde effects of botulinum neurotoxin A. J Neurosci. 2008;28(14):3689–96. 18385327 10.1523/JNEUROSCI.0375-08.2008 PMC6671090

[23] Willison HJ, Jacobs BC, van Doorn PA. Guillain-Barré syndrome. Lancet. 2016;388(10045):717–27. 26948435 10.1016/S0140-6736(16)00339-1

[24] Lonati D, Schicchi A, Crevani M, Buscaglia E, Scaravaggi G, Maida F, et al. Foodborne botulism: clinical diagnosis and medical treatment. Toxins (Basel). 2020;12(8): 509. 32784744 10.3390/toxins12080509 PMC7472133

[25] O’Horo JC, Harper EP, El Rafei A, Ali R, DeSimone DC, Sakusic A, et al. Efficacy of antitoxin therapy in treating patients with foodborne botulism: A systematic review and Meta-analysis of cases, 1923–2016. Clin Infect Dis. 2017;66(suppl1):S43–56. 29293927 10.1093/cid/cix815 PMC5850555

[26] Griese SE, Kisselburgh HM, Bartenfeld MT, Thomas E, Rao AK, Sobel J, et al. Pediatric botulism and use of equine botulinum antitoxin in children: A systematic review. Clin Infect Dis. 2017;66(suppl1):S17–29. 29293924 10.1093/cid/cix812

[27] Midura TF. Update: infant botulism. Clin Microbiol Rev. 1996;9(2):119–25. 8964030 10.1128/cmr.9.2.119 PMC172885

[28] Fagan RP, Neil KP, Sasich R, Luquez C, Asaad H, Maslanka S, et al. Initial recovery and rebound of type F intestinal colonization botulism after administration of investigational heptavalent botulinum antitoxin. Clin Infect Dis. 2011;53(9):e125–8. 21896700 10.1093/cid/cir550

[29] Jenzer G, Mumenthaler M, Ludin HP, Robert F. Autonomic dysfunction in botulism B: a clinical report. Neurology. 1975;25(2):150–3. 1167643 10.1212/wnl.25.2.150

[30] Patural H, Goffaux P, Paricio C, Emeriaud G, Teyssier G, Barthelemy JC, et al. Infant botulism intoxication and autonomic nervous system dysfunction. Anaerobe. 2009;15(5):197–200. 19327405 10.1016/j.anaerobe.2009.03.004

